# Polymerizable Choline- and Imidazolium-Based Ionic Liquids Reinforced with Bacterial Cellulose for 3D-Printing

**DOI:** 10.3390/polym13183044

**Published:** 2021-09-09

**Authors:** Michael A. Smirnov, Veronika S. Fedotova, Maria P. Sokolova, Alexandra L. Nikolaeva, Vladimir Yu. Elokhovsky, Mikko Karttunen

**Affiliations:** 1 Institute of Macromolecular Compounds, Russian Academy of Sciences, V.O. Bolshoi pr. 31, 199004 St. Petersburg, Russia; fedotova.veronicka2016@yandex.ru (V.S.F.); pmarip@mail.ru (M.P.S.); alexandra.l.nikolaeva@gmail.com (A.L.N.); Vladimir_Elokovskiy@hq.macro.ru (V.Y.E.); 2 Institute of Chemistry, Saint Petersburg State University, Universitetsky pr. 26, Peterhof, 198504 St. Petersburg, Russia; 3 Department of Chemistry, The University of Western Ontario, 1151 Richmond Street, London, ON N6A 5B7, Canada; 4 Department of Physics and Astronomy, The University of Western Ontario, 1151 Richmond Street, London, ON N6A 5B7, Canada; 5 The Centre of Advanced Materials and Biomaterials Research, The University of Western Ontario, 1151 Richmond Street, London, ON N6A 5B7, Canada

**Keywords:** bacterial cellulose, ionic liquids, 3D-printing, mechanical properties, ion gels

## Abstract

In this work, a novel approach is demonstrated for 3D-printing of bacterial cellulose (BC) reinforced UV-curable ion gels using two-component solvents based on 1-butyl-3-methylimidazolium chloride or choline chloride combined with acrylic acid. Preservation of cellulose’s crystalline and nanofibrous structure is demonstrated using wide-angle X-ray diffraction (WAXD) and atomic force microscopy (AFM). Rheological measurements reveal that cholinium-based systems, in comparison with imidazolium-based ones, are characterised with lower viscosity at low shear rates and improved stability against phase separation at high shear rates. Grafting of poly(acrylic acid) onto the surfaces of cellulose nanofibers during UV-induced polymerization of acrylic acid results in higher elongation at break for choline chloride-based compositions: 175% in comparison with 94% for imidazolium-based systems as well as enhanced mechanical properties in compression mode. As a result, cholinium-based BC ion gels containing acrylic acid can be considered as more suitable for 3D-printing of objects with improved mechanical properties due to increased dispersion stability and filler/matrix interaction.

## 1. Introduction

Manufacturing of products with complex shapes is a problem that has both practical and fundamental material-related challenges. The rise of 3D-printing based on biopolymer-based materials offers, however, a viable and versatile method for natural polymers [1,2,3,4]. Cellulose—the most abundant natural polymer—is a prospective candidate for the next generation of sustainable and biocompatible materials. Its crystalline parts demonstrate extremely high mechanical properties [5], which makes cellulose nanomaterials prospective reinforcing fillers [6,7,8,9,10]. Highly crystalline cellulose organized in nanofibrils can be produced by bacterial synthesis and is commonly referred to as bacterial cellulose (BC).

Due to the unique structure and chemical purity of BC, it has been studied as a prospective material for preparation of tough and strong fibers [11], scaffolds for cartilage tissue engineering [12], components for artificial cartilages [13] and as a good insulator composite material [14]. What complicates processing of cellulose is that its strong network of hydrogen bonds (hydrogen bonding in the context of cellulose in contact with membranes has been characterised e.g., in Refs. [15,16]) makes it insoluble in common solvents and non-meltable at temperatures lower than its decomposition temperature.

Ionic liquids (ILs) have melting points below 100 °C and demonstrate such attractive properties as high thermal [17] and chemical stability, low vapor pressure, structural diversity and miscibility with many other solvents [18,19,20,21]. ILs offer a potential solution to alleviate many of the problems and they have been applied in cellulose and biomass processing [22,23,24]. For example, they have been used for cellulose extraction from corn stover [25], in fractionation of lignocellulosic biomass [26], for the preparation of cellulose-based ionic porous materials [27] and conversion of cellulose into 5-hydroxymethylfurfural [28]. Moreover, cellulose-based composites with ILs can be obtained by radical polymerization, where imidazolium and choline acrylic derivatives act as monomers [29,30].

Over recent years, rheological properties of cellulose solutions in ILs have been studied intensely. This is particularly the case for imidazolium-based ILs. Viscosity and viscoelasticity are among the main properties of compositions used for 3D-printing of cellulose-based materials [31]. Continuous flow through the nozzles is achieved due to shear thinning, while shape preservation of the material after extrusion is provided by high storage modulus [32,33,34]. The details of viscous flow of cellulose solutions in ILs with concentrations from diluted up to concentrated regimes are attracting increasing attention and have been studied using 1-allyl-3-methylimidazolium chloride [35], 1-ethyl-3-methylimidazolium acetate [36,37,38] and 1-butyl-3-methylimidazolium chloride [36]. However, the use of imidazolium ILs is limited by their increased viscosity [39,40], which makes the use cellulose solutions in IL for 3D-printing difficult. This problem can be resolved by the addition of co-solvents [41,42]. For example, rheological properties of IL-cellulose formulations used for 3D-printing have been modified by the addition of dimethyl sulfoxide (DMSO) [43]. An important benefit of application of IL-based solvents is their ability to prevent the collapse of 3D-printed materials—a common problem in the case of water-based inks [31]. However, investigations regarding the application of ILs for 3D-printing of cellulose-based materials remain few. Among the disadvantages of ILs that limit their practical applications is the high cost [44].

A very promising cheaper and “green” subclass of ILs, deep eutectic solvents (DES), has been attracting tremendous interest over the recent years [45,46,47]. DES can be easily prepared from two components (hydrogen bond donor (HBD) and acceptor (HBA)) with melting temperature significantly below the melting temperatures of the individual components. Additionally, DES can be composed of components of natural origin, for example choline chloride, citric acid or glucose [48,49]. This makes them interesting for increasing sustainability in chemical technology. Due their capability to form hydrogen bond networks, DES have demonstrated outstanding ability to interact with polysaccharides [22] and they are being intensely studied as prospective plasticizers for chitosan [50,51,52,53], starch [54] and as media for treatment of chitin [55,56,57] or cellulose [58,59,60,61,62,63,64,65]. Additionally, using unsaturated acids such as acrylic or methacrylic acids as hydrogen bond donors [66] provides DES the ability to polymerize in a new green way for preparation of functional materials [67].

The main approaches for 3D-printing of materials with cellulose are fused deposition modeling [68], digital light processing [69] and robocasting [70] with or without subsequent UV-curing. Among the UV-curable components of materials for 3D-printing, acrylates have attracted the most attention because acrylic acid and its derivatives are capable of rapid photopolymerization [71,72]. For example, the use of water dispersions of cellulose nanocrystals mixed with 2-hydroxyethyl methacrylate [73], acrylic acid (AA) [71] and a mixture of AA with 3-dimethyl (methacryloyloxyethyl) ammonium propanesulfonate [74] for 3D-printing of cellulose-reinforced hydrogels has been reported. At the same time, composite ion gels based on cellulose and ILs are also considered as attractive materials with a wide range of prospective applications such as strain sensors [75], components for flexible electronic devices [76], preparation of a bactericidal hydrogel dressings [77] and air filters that have a great potential in air purification [78]. In approaches based on UV-curing, DES and ILs containing photo-polymerizable components can be proposed as a promising medium for preparation of cellulose-based viscous gels suitable for 3D-printing. However, DES-containing acrylic acid for direct 3D-printing of cellulose reinforced ion gel has been utilized only in the recent work of Lai and Yu [75], in which the ink was prepared using commercial cellulose nanocrystals, and DES based on choline chloride (ChCl) and ethylene glycol mixed with acrylic acid as UV-polymerizable monomer and Al_2_(SO_4_)_3_ as a cross-linker; ethylene glycol was used as the main HBD in DES. To the best of our knowledge, rheological properties and 3D-printing of cellulose dispersion in DES containing acrylic acid as the only HBD has not been reported yet. In order to fill this gap, we have studied two cases of polymerizable compositions for the preparation of cellulose nanofibers (CNF)-reinforced ion gels. The first one is based on IL 1-butyl-3-methylimidazolium chloride (ImCl) mixed with AA and the second one is a mixture of ChCl as a “greener” alternative for imidazolium-based salts and AA. Based on the method we have proposed for the preparation of CNF gel from BC using DES based on ChCl [79], here we suggest that mixtures based on organic chloride salt and acrylic monomer can be directly used as a media for the preparation of a CNF ion gel from BC. Furthermore, we also suggest that they are directly applicable for 3D-printing with subsequent UV-curing.

## 2. Materials and Methods

### 2.1. Materials

Lyophilized culture of *Acetobacter xylinum* was purchased form All-Russian collection of industrial microorganisms (National Bioresource Center, GosNIIgenetika, Moscow, Russia) and cultivated as described further. 1-butyl-3-methylimidazolium chloride (Sigma-Aldrich, Buchs SG, Switzerland, CAS 79917-90-1, purity > 98%) and choline chloride (Glentham Life Sciences Ltd., Corsham, UK, CAS 67-48-1, purity > 99%) were dried under vacuum at 60 °C for at least 24 h before use. N,N′-methylene-bis-acrylamide (Sigma-Aldrich, St. Louis, MO, USA, CAS 110-26-9, purity > 99.5%), acrylic acid (Sigma-Aldrich, CAS 67-48-1, purity > 99%) and 2-hydroxy-2-methylpropiophenone (Sigma-Aldrich, Milan, Italy, CAS 7473-98-5, purity > 97%) were used as received. Peptone and D-mannitol (CAS 69-65-8) were obtained from LenReaktiv (Saint Petersburg, Russia), yeast extract from Research Center for Pharmacotherapy (Saint Petersburg, Russia) and NaOH (CAS 1310-73-2) from NevaReaktiv (Saint Petersburg, Russia).

### 2.2. Methods

#### 2.2.1. Bacterial Synthesis of Cellulose

**Figure 1:**
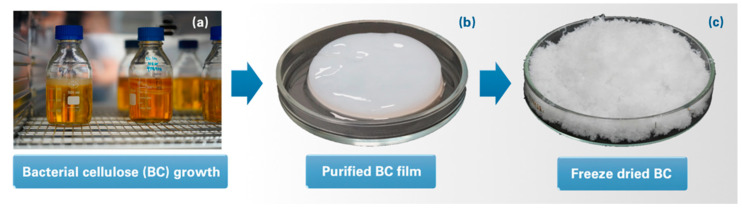
BC growing (**a**) a purified BC hydrogel (**b**) and BC after shredding and freeze drying (**c**).

The activation of lyophilized *A. xylinum* was carried out by introducing a seed culture medium. A single *A. xylinum* colony was transferred to 400 mL of seed culture medium and cultivated statically for 3 months at 27 °C (Figure 1). Then, 200 mL of the cell suspension was introduced into a bottle with 1 L of culture medium containing 0.3 wt% of peptone, 0.5 wt% of yeast extract and 2.5 wt% of D-mannitol. The medium was incubated statically at 27 °C for 15–20 days in an incubator (BINDER, Tuttlingen, Germany) until the formation of a BC membrane with a thickness of 1 cm. After that, the BC membrane was purified by boiling in 0.5 wt% NaOH solution for 30 days, and then thoroughly washed with distilled water until pH reached 7. After that, the membrane was mechanically ground, and the obtained suspension was diluted in order to prepare 0.5 wt% concentration of cellulose. The obtained mass was lyophilized with a Scientz-10ND lyophilic dryer (Scientz, Ningbo, China) and then ground into small pieces with 1–3 mm average size.

#### 2.2.2. Preparation of Dispersions and Regeneration of Cellulose

**Figure 2:**
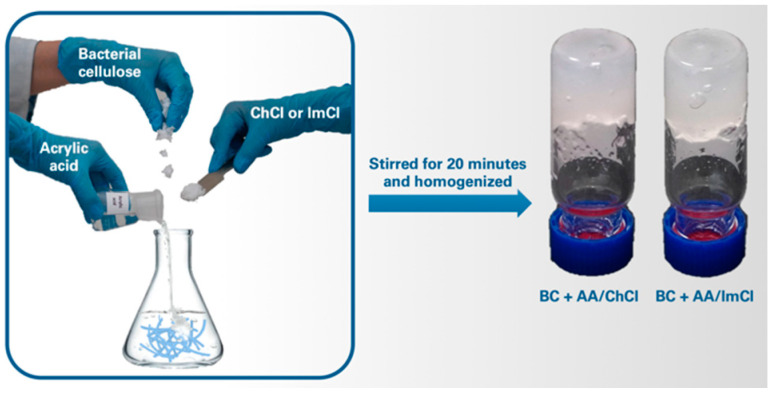
Preparation scheme and images of BC ion gels in AA/ChCl and AA/ImCl mixtures.

The solvents were prepared by mixing of ChCl or ImCl with AA in the mole ratio 1:3. For this purpose, 3.161 g of ChCl or 3.199 g of ImCl were mixed with 4.894 and 3.960 g of AA, respectively. The mixtures were stirred at 70 °C until a clear liquid formed. After that, lyophilized bacterial cellulose was added and the viscous mixture was stirred for 20 min at 20 °C and relative humidity of 40–45% until complete wetting of cellulose with the solvent. After that, the mixture was homogenized by multiple pushing of the mass through the die with 0.58 mm diameter. These systems (Figure 2) were used for rheological measurements and regeneration of cellulose with excess of water. The regenerated cellulose was precipitated with centrifugation for 20 min at 4000 rpm using Microprocessor Centrifuge (Digisystem Laboratory Instruments Inc., New Taipei City, Taiwan). In order to remove residual components of IL, the regenerated cellulose was redispersed in distilled water and centrifuged again; this was repeated 5 times.

#### 2.2.3. Microscopic Investigation

Atomic force microscopy (AFM) studies were performed using a SPM-9700HT scan- ning probe microscope (Shimadzu, Kyoto, Japan). The AFM images were taken in air at room temperature. The instrument operated in the force dynamic mode, and an NSG30_SS Silicon tip (tip curvature radius 2 nm) was employed. Images with 512 × 512 points were obtained.

#### 2.2.4. Wide-Angle X-ray Diffraction Study

Crystallinity of the samples was studied by wide-angle X-ray diffraction (WAXD) using a D8 Discover diffractometer (Bruker, Rheinstetten, Germany) equipped with a CuKa radiation source (λ = 1.54 Å). The WAXD patterns were studied in the 2θ range of 5° to 40°.

#### 2.2.5. Fourier Transform Infrared Spectroscopy

Attenuated total reflection (ATR)-FTIR spectroscopy study was performed on the IRAffinity-1S spectrometer (Shimadzu, Kyoto, Japan) with 100 scans at resolution 2 cm^−1^ from 3750 to 700 cm^−1^.

#### 2.2.6. Rheological Studies

Kinematic viscosities of the binary mixtures AA/ChCl and AA/ImCl were determined at 20 °C using a capillary viscometer with inner diameters of 0.99 and 0.73 mm (according to GOST 10028-81), respectively. Before the experiment, the viscometer constant was calibrated using a solution of water in glycerol (water content 20.85 and 30.45 wt%). Temperature control after filling the viscometer was carried out using LT-400 thermostat (LOIP, Moscow, Russia). The time of sample expiration through the capillary was recorded by a stopwatch. Kinematic viscosities were calculated by multiplying the expiration time by the viscometer constant and converted to dynamic viscosity using the density of systems.

Rheological studies were performed with the aid of a Physica MCR 301 rheometer (Anton Paar, Graz, Austria) equipped with cone-plate (20 mm diameter, 2° cone angle) geometry in shear and dynamic regimes at 20 °C.

#### 2.2.7. Polymerization in Bulk and 3D-Printing

**Figure 3:**
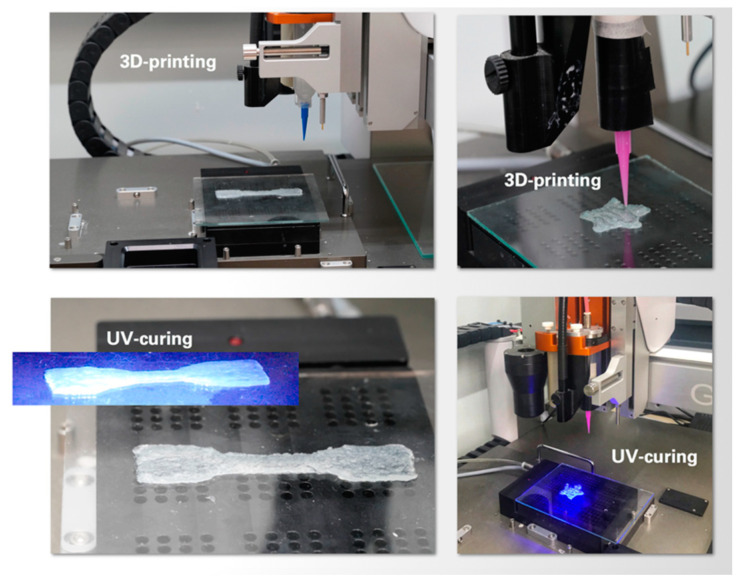
Examples of 3D-printed structures prepared using compositions based on AA/ChCl (dog-bone-shaped) and AA/ImCl (star).

The UV-curable compositions were prepared by the addition of 48.9 and 39.6 mg of bifunctional cross-linker (N,N′-methylene-bis-acrylamide), and 48.9 and 39.6 mg of photoinitiator (2-hydroxy-2-methylpropiophenone) were added, respectively, to the CNF dispersion based on AA/ChCl (8.137 g) and AA/ImCl (7.232 g). The amounts of cross-linker and initiator were chosen as 1 wt% from the mass of the polymerizable monomer (AA). The prepared compositions were used for UV-induced polymerization in polyethylene tubes for obtaining bulk material in the form of cylinders, or 3D-printed in the form of strips (Figure 3) in order to test the mechanical properties of filaments prepared via extrusion. For this purpose, the 3D-printer 3D BioScaffolder BS3.2 (GeSIM, Radeberg, Saxony, Germany) equipped with a pneumatic syringe with a 0.58 mm diameter nozzle was applied. UV-curing in all cases was performed using UV-lampOmniCure S1500 (Lumen Dynamics, Mississauga, ON, Canada) with light power 0.15 W cm^−2^. All samples (including polymerized in bulk and 3D-printed) were irradiated 7 times with 15 s and 20 s pauses between irradiations.

#### 2.2.8. Measurements of Mechanical Properties

An AG-100kNX Plus setup (Shimadzu, Kyoto, Japan) was used to study the mechanical characteristics. The measurements were performed in two testing modes. In the first one, the strip samples (with cross-section 1 × 5 mm^2^) were stretched at a rate of 50 mm/min, the gauge length being 15 mm. Experiments using the strip samples were performed according to the ASTM D882 requirements. Young’s modulus E, the break stress σ_b_, and the ultimate deformation ε_b_ were determined. The specimens were printed by aligning 4 filaments co-linearly with the direction of extension with subsequent UV-curing. The second testing mode consisted of a single-shot uniaxial compression of block cylinders at a rate of 2 mm/min up to 80% deformation. The so-called current compression moduli [13] E_10–15%_ = Δσ/Δε were determined in the deformation range of 10–15%, as well as stress values corresponding to 50 and 80% compression of the samples (σ_50%_ and σ_80%_), respectively. The latter were fabricated as follows: Photopolymerization of the compositions was conducted in the tube with the inner diameter of 9 mm by applying UV-irradiation. The obtained cylinder was cut into small ones with heights of about 5 mm. All the mechanical tests were performed at room temperature.

**Figure 4:**
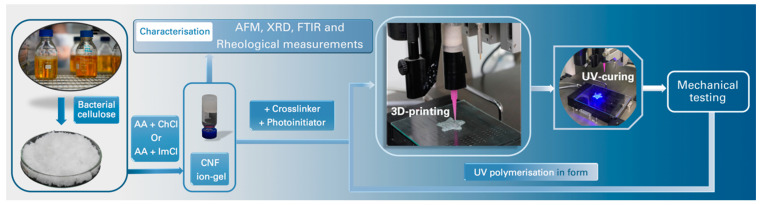
Block scheme demonstrating the preparation of materials and their characterization.

The design of materials preparation and investigation is summarized in Figure 4.

## 3. Results

### 3.1. Microscopy Investigation

**Figure 5:**
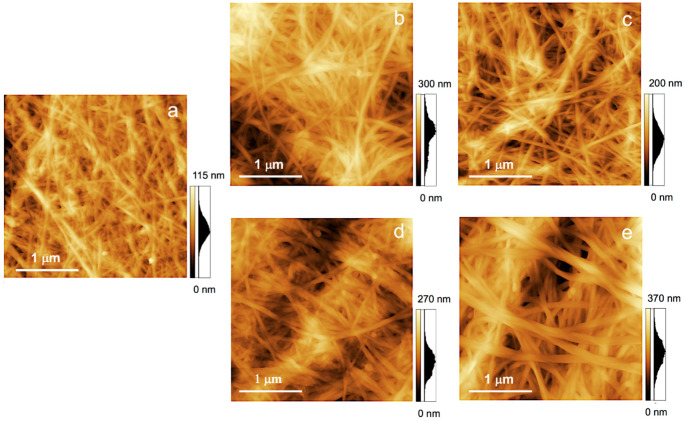
Surface topography of the initial BC (**a**), BC after AA/ImCl (**b**), after AA/ChCl (**c**), BC-PAA from AA/ImCl (**d**) and from AA/ChCl (**e**).

To evaluate the effects of AA containing ionic liquids on the morphology of BC, an AFM study was conducted. It was performed in two cases: (1) Only treatment with IL and (2) treatment with subsequent polymerization of AA. The height maps for the initial BC (Figure 5a), BC regenerated before polymerization from AA/ImCl (Figure 5b) and AA/ChCl (Figure 5c) demonstrate an approximately similar fibrous structure.

The profiles demonstrating the changing of fibrous diameter after different treatments are given in Figure S1. It is seen that BC nanofibers regenerated before polymerisation are thicker than in the initial BC sample: The diameter can be estimated to be 78–95 nm and 55–75 nm in samples regenerated from AA/ChCl and AA/ImCl, respectively, in comparison with 40–70 nm in the initial BC. The thickening effect is more pronounced in the case of the AA/ImCl solvent. This can be due to swelling of the cellulose fiber surfaces as a result of possible introduction of ImCl components between the cellulose macromolecules. This idea is supported by the earlier results [60,80] that demonstrate the swelling of cellulose nanofibers in the ChCl/imidazole mixture and 1-ethyl-3-methylimidazolium acetate, respectively. Nanofiber morphologies regenerated after AA polymerization from AA/ImCl and AA/ChCl are given in Figure 5d,e, respectively. The profiles (Figure S1) demonstrate that polymerisation of AA leads to an increase in nanofiber diameter up to 86–100 nm and 85–140 nm for AA/ImCl and AA/ChCl, respectively; the increase in thickness during polymerisation is more pronounced with the AA/ChCl solvent. Thickening during polymerisation can be attributed to possible grafting of poly(acrylic acid) to the cellulose surface, which was confirmed by FTIR measurements and will be discussed later in Section 3.3.

### 3.2. WAXD Study

**Figure 6:**
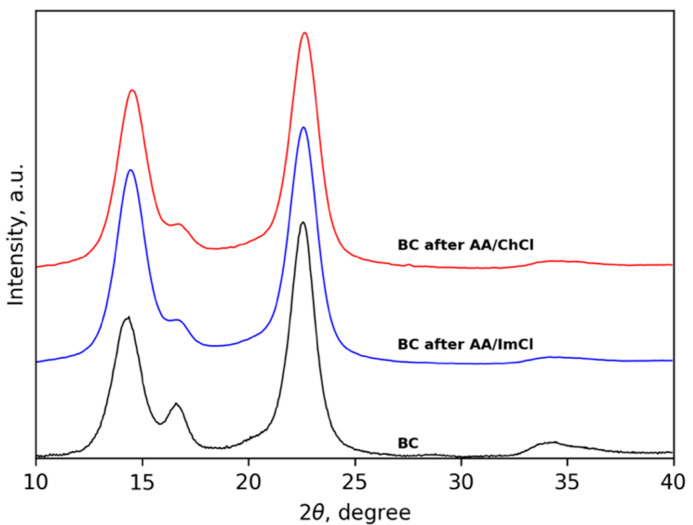
WAXD patterns of initial BC, BC after AA/ImCl and after AA/ChCl.

WAXD patterns of the initial BC, BC after AA/ImCl and after AA/ChCl are presented in Figure 6. Typical diffraction peaks are seen at 2θ = 14.8°, 16.6°, 22.6° and 34.4° corresponding to (1–10), (110), (200) and (004) lattice planes of the cellulose Iβ polymorph. The positions of reflections are preserved after treatment of BC with both types of used ILs. Using the Segal method [81], the degree of crystallinity ($\chi_{c}$) can be estimated as

(1) $$\chi_{c}=\frac{I_{200}-I_{am}}{I_{200}}\times 100$$

where *I_am_* and *I*_200_ are the diffraction intensities at 2θ = 18° and at the maximum of the (200) lattice diffraction plane. Using this equation, the values of $\chi_{c}$ were calculated as 96, 92 and 92% for the initial BC and CNF after treatment with AA/ChCl and AA/ImCl, respectively. Thus, the XRD results confirm the AFM data about preservation of the BC nanofibrilar structure during the treatment with AA/ChCl and AA/ImCl. The redistribution of intensities between the diffraction peaks at 2θ = 14.8° and 16.6° can be connected to the preferential orientations of CNF in dried films along the surface plane.

### 3.3. Fourier Transform Infrared Spectra

**Figure 7:**
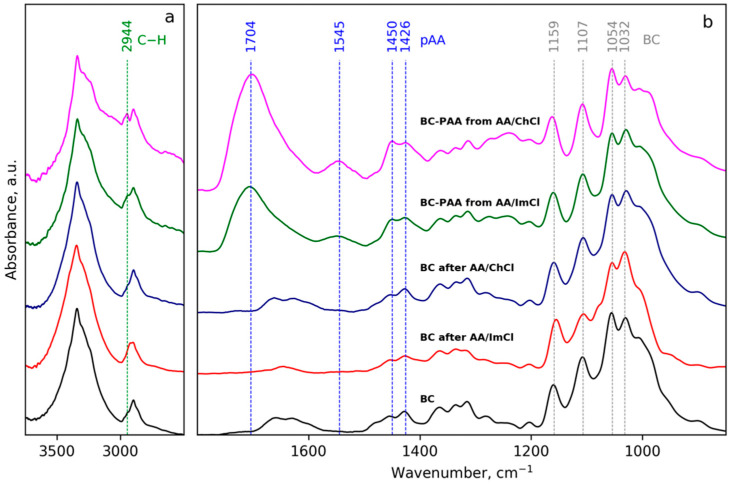
FTIR spectra of initial BC, BC after AA/ImCl and after AA/ChCl, BC-poly(acrylic acid) (BC-PAA) from AA/ImCl and from AA/ChCl in the regions 3700–2500 cm^−1^ (**a**) and 1800–900 cm^−1^ (**b**).

FTIR spectra were measured for the initial BC, for regenerated CNF from ionic liquids and for CNF purified after polymerization of the AA component with water (Figure 7). The results demonstrate that typical absorption bands of BC in the region of 1200–1000 cm^−1^ remain approximately at the same positions for all treated samples. That allows one to conclude that the chemical structure of the initial BC is not significantly affected. Samples regenerated from non-polymerized ILs (AA/ImCl and AA/ChCl) did not demonstrate significant changes in the FTIR spectra. At the same time, CNFs regenerated and purified via multiple repetitions of centrifugation/redispersion after polymerization of ILs (without cross-linker) demonstrate the appearance of intensive bands with maxima at 1704 and 1545 cm^−1^. These maxima can be attributed to the carboxylic groups in protonated and deprotonated forms, respectively.

Comparison of intensities of bands near 1054 and 1704 cm^−1^ (Figure 7) allows us to propose that in the case of AA/ChCl, the amount of grafted poly(acrylic acid) is higher than in the case of AA/ImCl. This observation is in agreement with the AFM data in Section 3.1. The appearance of grafted poly(acrylic acid) onto the CNF surface is also supported by the increase in absorption intensities near 2944 and 1450 cm^−1^, which can be attributed to the vibrations of the C–H bonds in the poly(acrylic acid) main chain [82].

### 3.4. Rheological Measurements

**Figure 8:**
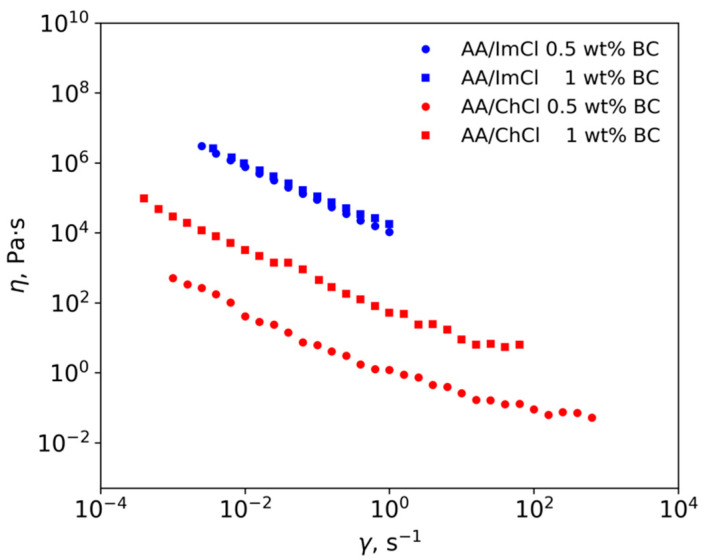
Steady-state viscosity (η) of the different compositions as a function of shear rate (γ) at 20 °C.

The dynamic viscosity of AA/ChCl at room temperature is higher than that of AA/ImCl: 56.7 and 24.5 mPa·s^−1^, respectively. The dependence of shear viscosity on the shear rate for ion gels containing BC (Figure 8) demonstrates shear thinning in all systems and concentrations. In the case of dispersions in AA/Im, no plateau at high shear rates was achieved because of the instability at shear rates higher than 1 s^−1^. In the case of ChCl-based systems, the measurements gave stable results up to 63 s^−1^ and 1000 s^−1^ for dispersions with 1 and 0.5 wt% of CNF, respectively. The appearance of a plateau at the high shear rate region with viscosities about 6.3 Pa·s^−1^ and 0.05 Pa·s^−1^ was observed for dispersions with 1 and 0.5 wt% of CNF, respectively. The flow instabilities are connected to solid-like behavior of gels, as will be demonstrated further.

CNF gels in AA/Im have higher viscosities in comparison with dispersions in AA/ChCl. The AFM results (Figure 5) demonstrate that CNF regenerated from AA/ImCl have slightly higher diameters in comparison with the AA/ChCl systems. The swelling of the CNF surface layer in imidazolium-based solvents possibly leads to an increasing amount of surface −OH groups being accessible for an interaction with solvent and between the CNFs. This leads to the formation of a stronger hydrogen bond network in the case of CNF gels in AA/ImCl in comparison with the AA/ChCl-based systems.

**Figure 9:**
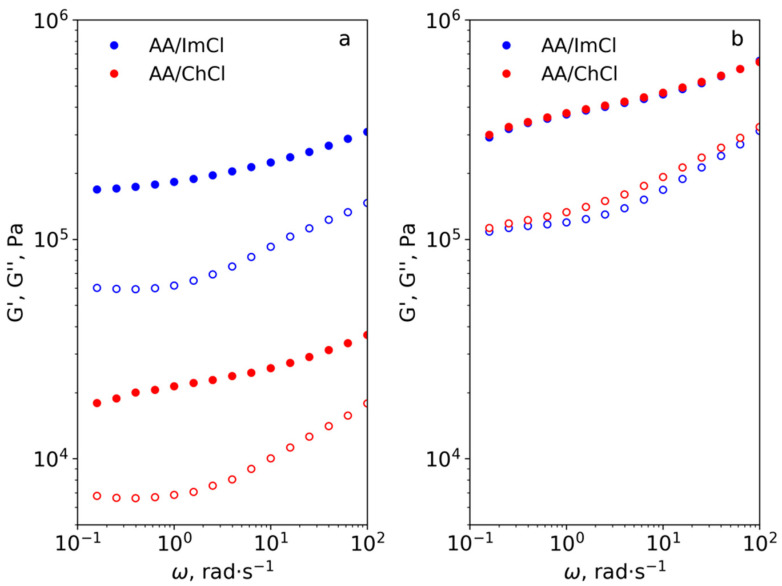
Storage (G’, filled symbols) and loss (G′′, hollow symbols) moduli as a function of angular frequency (ω) for dispersions containing 0.5 wt% (**a**) and 1 wt% (**b**) of CNF at 20 °C.

The results of viscosity measurements in the dynamic regime are given in Figure 9. The higher G’ (filled symbols)values in comparison with G’’ (hollow symbols) for all studied systems demonstrate the gel-like behavior of the material with predominantly elastic properties. This implies the formation of a strong CNF network even at the smallest cellulose concentration in dispersions. Taking this into account, it can be proposed that instability in the rheological measurements in shear mode can be connected to pronounced elastic behavior. The lack of viscous properties can result in the disruption of the CNF network at high shear rates with a sharp drop in mechanical properties. The lesser influence of CNF concentration on G’ and G’’ in AA/ImCl systems in comparison with AA/ChCl-based systems (difference between Figure 9a,b) is in agreement with results of viscosity measurements in shear mode. This demonstrates that in the case of AA/ImCl, a strong percolation network of CNF is formed even at 0.5 wt% and increasing the concentration up to 1 wt% leads to a two-fold increase of the G’ modulus. In the case of AA/ChCl, similar changes in CNF concentration lead to the growth of G’ by an order of magnitude. In the case of 1 wt% of CNF, the values of G’ and G’’ are approximately equal for both studied solvents. This means that the mechanical properties at small deformations are determined only by the mechanics of CNF. At the same time, the viscosities of these systems in shear mode are different (Figure 8) demonstrating easier rearrangement of the CNF network in the case of AA/ChCl.

### 3.5. 3D-Printing and Mechanical Properties

Compositions with 1 wt% of BC nanofiber content were chosen for 3D-printing. Before performing 3D-printing, the dependence of extrusion weight on the extrusion pressure was measured (Figure S2). The minimum pressure needed for extrusion was 20 kPa for both compositions. The extrusion weight for AA/ImCl was higher than in the case of AA/ChCl, which is in agreement with rheological measurements using pure solvents. However, the dependence of extrusion weight on the type of cation is in the opposite direction with steady-state rheological viscosity of ILs containing BC (Figure 8) as will be discussed further.

**Figure 10:**
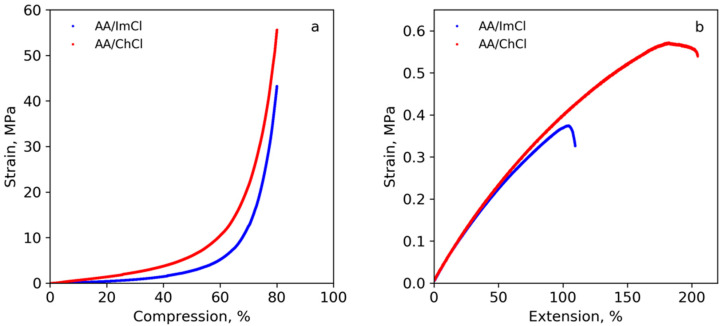
Stress–strain curves of AA/ChCl- and AA/ImCl-based samples prepared by polymerization in bulk and measured in compression mode (**a**) and prepared via 3D-printing and measured in extension mode (**b**).

The mechanical properties measured in compression and extension modes are summarized in Table 1 and Table 2, and the corresponding stress–strain curves are given in Figure 10a,b. Figure S3 shows images of initial and stretched ion gel under mechanical testing.

When analyzing mechanical properties of polymerized samples in compression mode, one should take into account that because of the nonlinearity of the stress–strain curves at small deformations, it is more appropriate to characterize the stiffness of such objects by the so-called current compression modulus E_10–15%_ = Δσ/Δε in the deformation range of 10–15% (Table 1) instead of elastic modulus [13]. Figure 10a shows that samples based on both compositions are able to withstand high compression fairly well, demonstrating no visible signs of destruction even at 80% deformation. A slow increase was observed for deformations up to about 40–50% followed by a steep increase in the compression stress at higher deformations. It is noteworthy that both E_10–15%_ and σ_50%_ for the AA/ChCl-based sample are substantially higher than those for the AA/ImCl-based one (Table 1). This is probably related to the higher degree of grafting of growing poly(acrylic acid) onto the cellulose nanofiber surfaces during UV-induced radical polymerization as it was shown earlier with AFM and FTIR measurements. This process can lead to stronger interactions between reinforcing filler (cellulose nanofibers) and matrix–crosslinked poly(acrylic acid) network and chloride ions via additional hydrogen bonding.

It is evident from Figure 10b and Table 2 that both types of specimens, namely those fabricated from either AA/ChCl or AA/ImCl via 3D-printing, are quite soft and characterized by an elastic modulus of about 0.5 MPa. Replacing ImCl with ChCl in the polymerizable composition led to an enhancement of mechanical properties, with both strength and ultimate deformation of AA/ChCl-based samples being about 1.5 times higher than those of the AA/ImCl-based ones.

## 4. Discussion

In this work, viscous nanofibrous ion gels were prepared via dispersion of bacterial cellulose in two types of ionic liquids: (1) Based on 1-butyl-3-methylimidazolium chloride or (2) choline chloride. UV-curing was achieved due to using acrylic acid and a bifunctional cross-linker in the compositions. Structures were characterized by three main methods, AFM, WAXD and FTIR. The WAXD study demonstrates that the crystalline structure of the polymer remained unchanged. Additionally, the absence of noticeable scattering from amorphous parts demonstrates a high degree of crystallinity of both the initial and treated materials.

Together, the AFM and WAXD results prove that cellulose’s crystalline and fibrillar structures remain preserved under treatment with the given solvents. More pronounced swelling of nanofibers in the presence of 1-butyl-3-methylimidazolium chloride was observed, demonstrating the increased possibility of 1-butyl-3-methylimidazolium chloride to interact with cellulose. FTIR results further demonstrated the grafting of poly(acrylic acid) onto the surface of cellulose nanofibers in the course of UV-induced radical polymerization. This observation suggests that the CNF surface becomes modified during photo-initiated AA polymerization in choline- or imidazolium-based Ils [82]. The appearance of strong adsorption near 1700 cm^−1^ has been reported in the literature as evidence for grafting of PAA to starch [83], while those at 1557 and 1451 cm^−1^ are evidence of grafting of poly(sodium acrylate) onto carboxymethylcellulose [84]. Such a process can occur due to the formation of different types of free radicals on the cellulose molecules under UV-irradiation [85] in the presence of a photosensitizer (2-hydroxy-2-methylpropiophenone). This process is more pronounced for choline-based compositions. Furthermore, the higher viscosity of deep eutectic solvents has been proposed as one of the factors leading to an enhancement of the radical polymerisation rate [86], and this can be the reason for the higher degree of grafting of the AA/ChCl solvent in our case. Additionally, the lower accessibility of the cellulose surface for acrylic acid in the case of the 1-butyl-3-methylimidazolium chloride-based solvent can be speculated, but this needs further evaluation and is beyond the scope of the current study. Partial deprotonation of poly(acrylic acid) can be attributed to the possible capture of cholinium or imidazolium cations onto the surface-grafted acidic polymer brush with the formation of salts between acrylic acid and imidazolium or cholinium cations.

The higher viscosity of pure AA/ChCl in comparison with AA/ImCl can be explained by the following: As discussed above, high viscosity of ILs and DES is connected with the formation of hydrogen bond networks [87]. In the case of ChCl, mixing with AA leads to the formation of an ionic liquid due to the network of hydrogen bonds between the −COOH group of AA and the −OH groups of choline with Cl^−^ [88,89]. In the case of ImCl, the ionic liquid appears due to the formation of network-like interactions between Im and Cl^−^, and in this case, AA can be considered as a co-solvent, which destructs the network [90] thus reducing viscosity [91].

On the rheological side, the obtained dispersions exhibit shear thinning behavior, which makes them amenable for 3D-printing. This effect is due to the orientation of the CNFs in the direction of flow and slip on the rheometer wall since phase separation from the dispersed cellulose results in lubrication of the contact between the gel and the rheometer wall [92]. The dispersant can be depleted from the vicinity of walls by the presence of large particles [93], which in our case can be aggregates of CNF or CNF itself because their length can be longer than 3 μm. It has been reported earlier that hydrogels based on cellulose nanocrystals also exhibit shear thinning behavior [31]. However, it is worth noting that high viscosity at low shear rates (in our work, 2.6 × 10^6^ and 7976 Pa·s^−1^ for AA/ImCl and AA/ChCl, respectively) can be achieved with a large content of cellulose nanocrystals (10–25 wt%) [73,75] or by using additional polymers such as poly(ethylene glycol) hexadecyl ether and poly(ethylene glycol) diacrylate [94]. High viscosity at low shear rates is necessary to prevent material leakage from the nozzle during 3D-printing [31]. In our work, the use of CNF allows obtaining dispersions with a suitable viscosity at low shear rates even at 1 wt% of cellulose content. Comparing our results with the reported rheological properties of cellulose solutions in pure ILs, it is worth noting that shear thinning is either not observed, for example, in the case of 1-ethyl-3-methylimidazolium acetate [37] or 1-butyl-3-methylimidazolium chloride [38], or it is observed at high shear rates, as was demonstrated in the case of 1-allyl-3-methylimidazolium chloride [35]. This makes it difficult to use such systems for 3D-printing.

The viscosity of ion gels based on ImCl is higher than based on ChCl; that is, opposite to the viscosity of pure solvents. This observation can potentially be explained as follows: The less viscous solvent containing the more polarizable imidazolium cations can result in the formation of more dispersed cellulose because the more polarizable imidazolium cations are better for cellulose stabilization in solution in comparison with ternary ammonium cations [95,96]. Additionally, the π-electron delocalization of the unsaturated heterocyclic ring makes the cations more active in interacting with cellulose [97]. Thus, a higher number of entanglements between the CNFs will result in higher viscosity. In the case of 1-butyl-3-methylimidazolium chloride, bacterial nanocellulose dispersions demonstrate higher viscosity in comparison with choline-based systems at low shear rates. However, the higher flow stability at high shear rates for dispersions in choline chloride/acrylic acid solvent makes them more suitable for 3D-printing. The flow instabilities can be discussed on the basis of results of rheological measurements in the dynamic mode that implies the formation of a strong CNF network even at the smallest cellulose concentration in dispersions. Taking this into account, it can be proposed that the instability in the rheological measurements in the shear mode can be connected to pronounced elastic behavior. The lack of viscous properties can result in the disruption of the CNF network at high shear rates with a sharp drop in mechanical properties.

The dependence of extrusion weight on the type of cation correlates with viscosities of pure solvents: Higher rates were observed for AA/ImCl-based composition. This allows us to propose that during extrusion, flow is strongly affected by slippage near the walls of the nozzle. As a result, the lower viscosity of the AA/ImCl solvent leads to higher flow rates for BC dispersion in this solvent in comparison with AA/ChCl as can be seen from Figure S2. The wider error bars for BC in AA/ImCl (Figure S2) demonstrate lower flow stability for this system. This is in agreement with limits for steady-state viscosity measurements discussed earlier.

In our study, structural characterization was complemented by studies of mechanical properties. The measurements of 3D-printed strips in the extension mode showed that choline-based ion gels have higher elongation at break: 175% in comparison with 94% for imidazolium-based systems. Additionally, choline-based ion gels demonstrate a higher compression modulus and strength at 50% of compression. Considering the data on structural and rheological properties of the polymerizable mixtures discussed above, one could infer that higher mechanical properties might be associated with higher stability of ChCl-based dispersions under shear stress. Taking into account the assumption that phase stratification near the wall of the nozzle proceeds more intensely in the case of imidazolium-based system, formation of a more defective CNF network structure in the 3D-printed material based on this cation can be proposed. Additionally, the increased interaction between the filler and the matrix in AA/ChCl-based material can be proposed due to more pronounced grafting of poly(acrylic acid) onto the cellulose surface. This result is very interesting, since it demonstrates the higher properties for a “greener” composition.

The values of Young’s modulus reported in this work were 0.51 and 0.56 MPa for the 3D-printed samples based on AA/ImCl and AA/ChCl, respectively. These values are higher than those reported in Ref. [75] for similar systems based on cellulose nanocrystals (22.5 wt%) in mixtures of choline chloride and ethylene glycol containing acrylic acid (about 0.17 MPa). This can be connected to the use of a covalent cross-linker N,N′-methylenebisacrylamide, in our case in comparison with the use of reversible ionic cross-links in the work of Lai and Yu [75]. On the other hand, covalent cross-links provide lower values of elongation at break for samples prepared in this work: 94 and 175%, respectively, for AA/ImCl and AA/ChCl-based ion gels in comparison with 1100% reported in [75]. Because of the high concentration of the polymerizable monomer, the tensile strength in our case was also significantly greater than that in ChCl-urea-glycerol-based ion gels reinforced with 4 wt% of cellulose nanocrystals and obtained via polymerization of acrylamide with MBA as a cross-linker [98]: 55.5 kPa in comparison with 0.56 MPa for the AA/ChCl-based material in our case.

## 5. Conclusions

To conclude, in this work, we prepared UV-curable bacterial cellulose ion gels using two systems, choline- and imidazolium-based ionic liquids containing acrylic acid. First, and importantly, AFM and WAXD data demonstrate that the nanofibrous and crystalline structures of bacterial cellulose are preserved. In addition, the nanofibers swelled more in the presence of 1-butyl-3-methylimidazolium chloride.

Second, both compositions showed shear thinning behavior, making them amenable for 3D-printing. At the same time, the results from dynamic rheological measurements demonstrate the predominantly elastic behavior for all compositions (G′ > G′′) that can lead to instability in their viscous flow. The dispersions showed, however, higher resistance toward phase separation at high shear rates in choline chloride/acrylic acid solvent, thus making the choline-based composition more suitable for 3D-printing of the two.

Finally, FTIR data suggest more pronounced grafting of poly(acrylic acid) onto the surfaces of bacterial cellulose nanofibers during UV-curing in choline chloride/acrylic acid mixtures. Consequently, this leads to stronger interactions between the matrix and filler and yields higher mechanical properties. As a result, the “greener” choline-based ion gels demonstrate a higher compression modulus and strength at 50% of compression as well as higher elongation at break: 175% in comparison with 94% for imidazolium-based systems. In summary, a novel approach based on using mixtures of ionic liquids with acrylic acid for the preparation of bacterial cellulose-reinforced ion gel with subsequent 3D-printing and UV-curing has been demonstrated.

## Figures and Tables

**Table 1:** Mechanical properties of AA/ChCl- and AA/ImCl-based polymerized ion gels measured in compression mode for samples prepared by bulk polymerization.

Sample	E_10–15%_, MPa	σ_50%_, MPa	σ_80%_, MPa
AA/ImCl-based ion gel	2.1 ± 0.2	2.7 ± 0.3	45 ± 15
AA/ChCl-based ion gel	6.8 ± 0.9	5.7 ± 0.4	49 ± 6

**Table 2:** Mechanical properties of AA/ChCl- and AA/ImCl-based polymerized ion gels measured in extension mode for samples prepared via 3D-printing.

Sample	E, MPa	σ_b_, MPa	ε_b_, %
AA/ImCl-based ion gel	0.51 ± 0.05	0.38 ± 0.05	94 ± 14
AA/ChCl-based ion gel	0.56 ± 0.06	0.56 ± 0.02	175 ± 25

## Data Availability

Data available within the article or its supplementary materials.

## Supplementary Materials

The following are available online at https://www.mdpi.com/article/10.3390/polym13183044/s1, Figure S1: Profiles extracted from surface topography maps of initial BC (a), BC after AA/ImCl (b) and after AA/ChCl (c), BC-PAA from AA/ImCl (d) and from AA/ChCl (e), Figure S2: Dependences of extrusion weight on extrusion pressure for compositions containing 1 wt% of BC through nozzle with diameter of 0.58 mm. Each point was measured 3–5 times. Figure S3: Images of ion gel stretched in a mechanical testing machine.
