# Supplementary material for: Polymerizable Choline- and Imidazolium-Based Ionic Liquids Reinforced with Bacterial Cellulose for 3D-Printing
Source: Polymers (Basel). 2021 Sep 9;13(18):3044. doi: 10.3390/polym13183044 (PMC8471885; doi:10.3390/polym13183044)
Supplement: Supplementary file 1 [file polymers-13-03044-s001.zip › polymers-1356311-supplementary.pdf]

ELECTRONIC SUPPLEMENTARY INFORMATION FOR

Polymerizable Choline- and Imidazolium-Based Ionic Liquids Reinforced with Bacterial Cellulose for 3D-Printing

Michael A. Smirnov [1,2,*], Veronika S. Fedotova [1,2], Maria P. Sokolova [1], Alexandra L. Nikolaeva [1], Vladimir Yu. Elokhovsky [1] and Mikko Karttunen [1,3,4,5,*]

[1] Institute of Macromolecular Compounds, Russian Academy of Sciences, V.O. Bolshoi pr. 31, 199004 St. Petersburg, Russia
[2] Institute of Chemistry, Saint Petersburg State University, Universitetsky pr. 26, Peterhof, 198504 St. Petersburg, Russia
[3] Department of Chemistry, The University of Western Ontario, 1151 Richmond Street, London, ON N6A 5B7, Canada
[4] Department of Physics and Astronomy, The University of Western Ontario, 1151 Richmond Street, London, ON N6A 5B7, Canada
[5] The Centre of Advanced Materials and Biomaterials Research, The University of Western Ontario, 1151 Richmond Street, London, ON N6A 5B7, Canada
* Correspondence: Smirnov_Michael@mail.ru (M.A.S.); mkarttu@uwo.ca (M.K.)

1. AFM Measurements

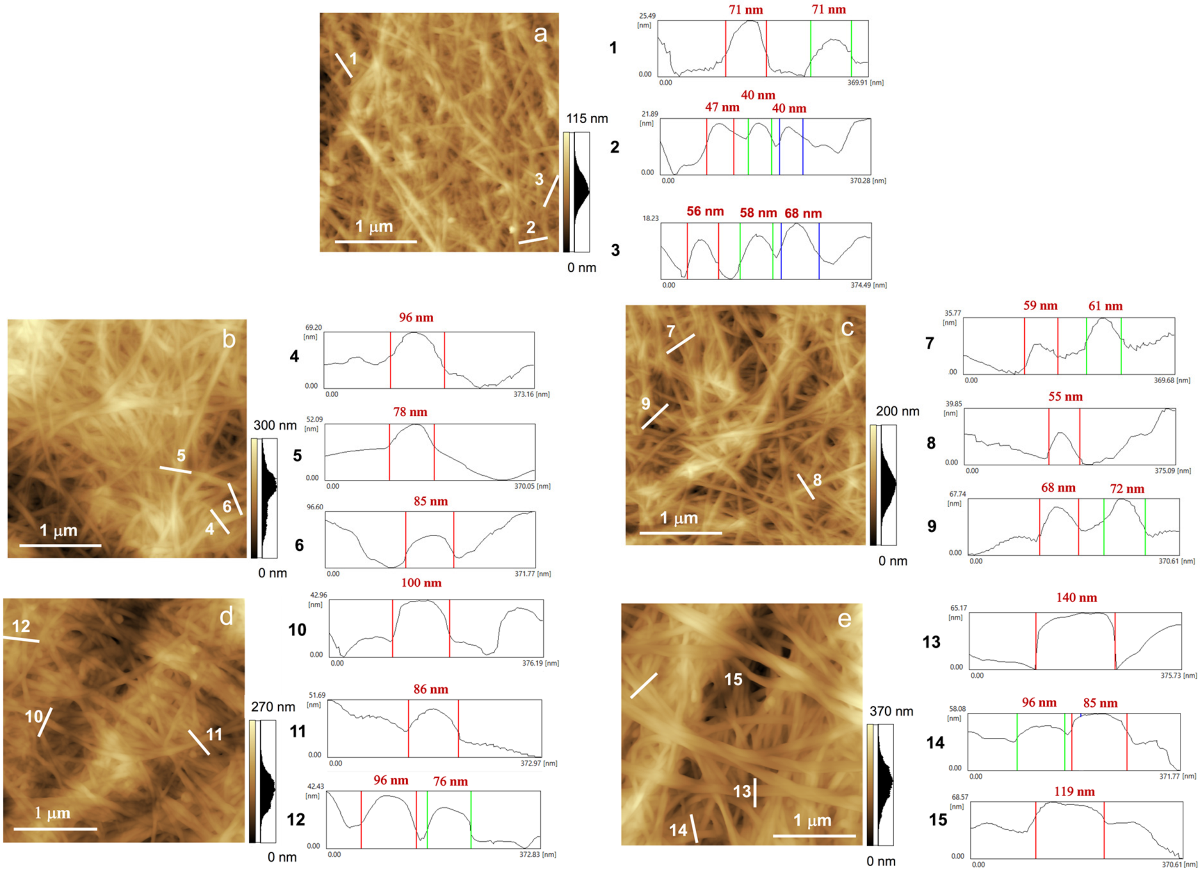

Figure S1. Profiles extracted from surface topography maps of initial BC (**a**), BC after AA/ImCl (**b**) and after AA/ChCl (**c**), BC-PAA from AA/ImCl (**d**) and from AA/ChCl (**e**).

2. Extrusion of CNF through Nozzle

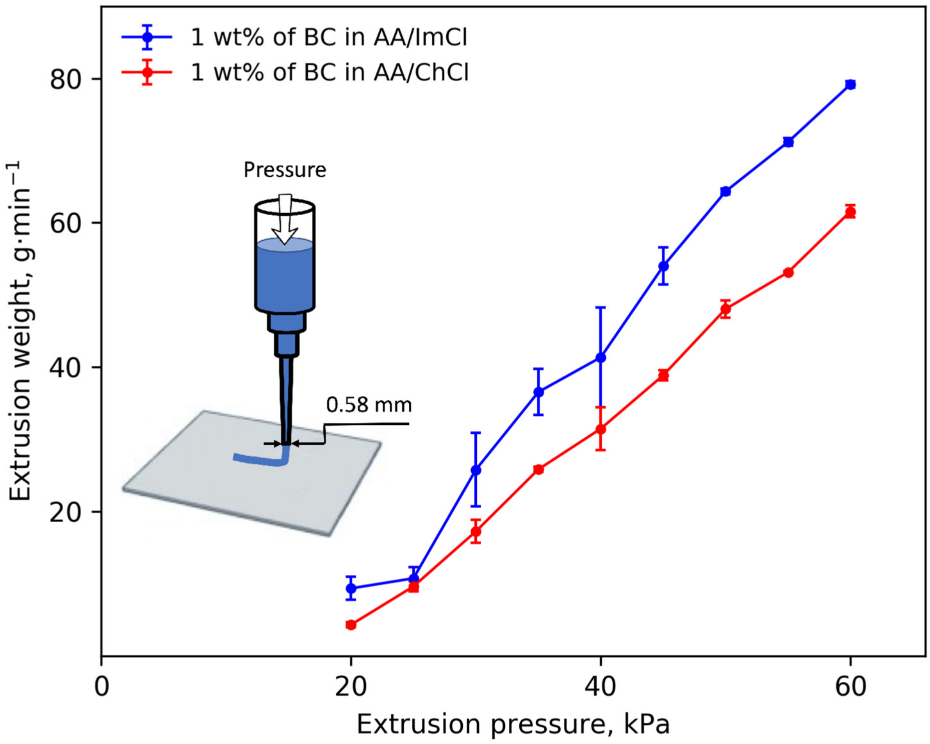

Figure S2. Dependence of extrusion weight on extrusion pressure for compositions containing 1 wt% of BC through nozzle of diameter of 0.58 mm. Each point was measured 3-5 times.

3. Mechanical Properties

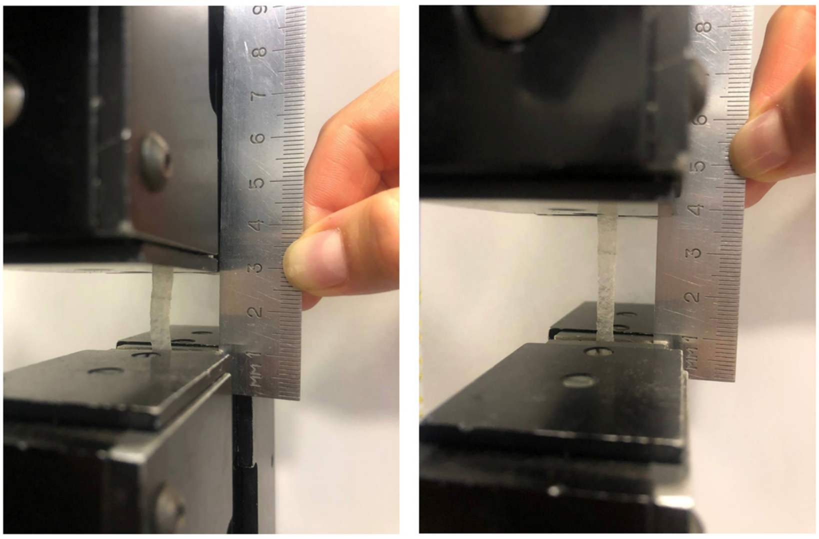

Figure S3. Images of ion gel stretched in mechanical testing machine.
